# Multimodal control of neck muscles for vestibular mediated head oscillation damping during walking: a pilot study

**DOI:** 10.1007/s00405-020-06488-5

**Published:** 2020-12-15

**Authors:** Matthias Hölzl, Winfried Neuhuber, Olaf Ueberschär, Axel Schleichardt, Natalie Stamm, Christoph Arens, Andreas Biesdorf, Ulrich Goessler, Roland Hülse

**Affiliations:** 1 Department of Otorhinolaryngology, Head and Neck Surgery, University Hospitals Otto-von-Guericke-University Magdeburg, Leipziger Str. 44, 39120 Magdeburg, Germany; 2 ENT Centre of Traunstein, Maxplatz 5, 83278 Traunstein, Germany; 3 Institute of Anatomy and Cell Biology, Friedrich-Alexander-University of Erlangen-Nürnberg, Krankenhausstraße 9, 91054 Erlangen, Germany; 4 Department of Engineering and Industrial Design, Magdeburg-Stendal University of Applied Sciences, Breitscheidstraße 2, 39114 Magdeburg, Germany; 5 Department of Biomechanics, Institute for Applied Training Science, Marschnerstraße 29, 04109 Leipzig, Germany; 6 Siemens AG, Technology, Otto-Hahn-Ring 6, 81739 München, Germany; 7 Department of Medicine in Physiotherapy, Faculty of Therapeutic Sciences, Maria-Probst-Str. 3, 69123 Heidelberg, Germany

**Keywords:** Vestibulocollic reflex (VCR)/cervicocollic reflex (CCR), Head stabilization, Neck muscles, Medial vestibulospinal tract (MVST), Cervical vestibular-evoked myogenic potential (cVEMP)

## Abstract

**Purpose:**

It is still in question whether head oscillation damping during walking forms a part of the vestibular function. The anatomical pathway from the vestibular system to the neck muscles via the medial vestibulospinal tract (MVST) is well known but there is a lack of knowledge of the exact influence and modulation of each other in daily life activities.

**Methods:**

(I) We fixed a head–neck unit of a human cadaver specimen in a steal frame to determine the required pitch-torque for a horizontal head position. The mean value of the acquired pitch-torque was 0.54 Nm. (II) On a motorized treadmill we acquired kinematic data of the head, the sternum and both feet by wireless 3D IMUs for seven asymptomatic volunteers. Subsequently three randomized task conditions were performed. Condition 1 was walking without any irritation. Condition 2 imitated a sacculus irritation using a standardized cVEMP signal. The third condition used an electric neck muscle-irritation (TENS). The data were analyzed by the simulation environment software OpenSim 4.0.

**Results:**

8 neck muscle pairs were identified. By performing three different conditions we observed some highly significant deviations of the neck muscle peak torques. Analysing Euler angles, we found during walking a LARP and RALP head pendulum, which also was strongly perturbated.

**Conclusion:**

Particularly the pitch-down head oscillation damping is the most challenging one for neck muscles, especially under biomechanical concerns. Mainly via MVST motor activity of neck muscles  might be modulated by **vestibular** motor signals. Two simultaneous proprioceptor effects might optimize head oscillation damping. One might be a proprioceptive feedback loop to the vestibular nucleus. Another might trigger the cervicocollic reflex (CCR).

## Introduction

The upright gait generates a continuous, rhythmic oscillation of the head. Daily activities require a sufficient head damping control and feedback system, which is not yet well understood.

Our research aims to introduce some basic vestibular considerations that also integrate biomechanical and muscular aspects to the head damping system. In relation to the control and feedback system of the head damping system, we investigate separately whether changes in head damping are caused by artificial irritation of the sacculus disturbing the control mechanisms or by muscle irritation disturbing the feedback mechanisms.

Using a pilot study, volunteers walked in three different walking conditions on a motorised treadmill. The dynamic data from inertial sensors were processed in the OpenSim software [1, 2].

Due to the anatomical asymmetry only the pitch plane (head flexion/extension) in the human head–neck unit, we need better knowledge about their position of the centre of gravity (CG). A gap between CG and an axis of rotation is a highly relevant parameter for all damping systems in general.

Additionally, therefore, we examined in pitch plane the CG of one entire head–neck unit from the head to the segment C5/6 on an anatomical specimen.

Defining the maximum torques of the most relevant neck muscles may be a key parameter in understanding head stability during a walking cycle.

## Materials/methods

### Aspects of head damping biomechanics

The head of an adult body donated to the Institute of Anatomy at the University of Erlangen-Nürnberg for teaching and research purposes was separated from the trunk at level C5/6. The head was fixed in a custom-made steel frame (Junghans^©^, GER) by transverse pins along its pitch axis (through the anterior edge of the mastoid process, slightly above external acoustic meatus, according to Kapandji [3]) thus allowing free rotation. The force (N) required to keep the head/neck unit in the horizontal position was determined using a spring scale hooked into a small skin incision in the occipital scalp of the specimen (Fig. 3). The length of the lever was 6 cm. The average of three measurements were used to calculate the torque.

### Aspects of the head damping control and feedback system

#### Data acquisition

In a pilot study setup, we enrolled seven middle-aged asymptomatic volunteers (mean age 42.6 years, SD ± 6.7) to walk on a motorised treadmill (Kettler Track 9) at 0.7 m/s. Macroscopic body segment kinematics were acquired using triaxial magneto-inertial measurement units (IMUs). Those datasets included triaxial acceleration vectors (in the IMU’s local frame of reference) and Euler angles, as derived from microelectronic triaxial accelerometer, magnetometer and gyroscope measurements at a data rate of 25–100 Hz [Bonsai-Systems (www.bonsai-systems.com)]. Euler angles are three angles that allow a description and orientation of a rigid body in a three-dimensional space or coordinate system. Those IMUs were fixed noninvasively onto the head (centre of the sagittal suture), the feet (dorsally, third metatarsal) and thorax (anteriorly, sternum). The degrees of freedom of the cervical spine in terms of neck flexion/extension (expressed in the cranial pitch angle), lateral flexion/extension (roll) and transversal rotation (yaw) were assigned to their dominant localisations in the C0/C1 and the C2–7/Th1 vertebral joints, respectively. The corresponding articular movements were calculated as the difference between the sternum and head Euler angles for each spatial axis as an angular time series. All raw data were processed by customised routines calculated in GNU Octave 4.0.

To enter the pilot-study, the volunteers required normal results in a complete neurovestibular investigation test setup including caloric, VEMP, vHIT and subjective visual vertical (Laser adapted bucket test) testing. As exclusion criteria, we defined normal results in the neck disability index (NDI) and the dizziness handicap inventory (DHI) questionnaires. The age of the volunteers has to be older than 18 and younger than 50 years. Another criterion for exclusion were any relevant orthopaedic or neurologic diseases in patient history.

On the treadmill, the volunteer was instructed to fixate on a point at a distance of 50 cm. To separate aspects of head damping’s control and feedback system, we create a standardised test setup. Each volunteer performed three different walking conditions for 3 min. A short reset period followed each condition.

1. Gait without any external influences.
2. Gait with perturbation of the vestibular system by acoustic sacculus irritation via headset (cVEMP stimulus of Interacoustics^©^ DN).
3. Gait with perturbation of the neck muscle system by electric stimulation via a transcutaneous applicated electrical neck muscle stimulation (monophasic rectangular pulse 150 μs, frequency 20 Hz, intensity 100 mA; transcutaneous electric nerve stimulation (TENS) of Schwa-Mediko© GER).

### Simulation-based force and torque analyses

Head and neck muscle characteristics, including muscle-specific maximal isometric contraction forces and contraction velocities, were adopted from the established biomechanical human neck model of Mortensen and colleagues [4]. To obtain quantitative figures on inner muscle forces, was calculated within the simulation environment OpenSim 4.0 [1, 2]. Muscle lengths, however, were scaled linearly to fit the subject’s individual stature. For each subject and experimental setting, 10 s of treadmill walking was analysed after discarding 30 s of initial gait familiarisation. The resulting time series of muscle forces and moment arms were statically processed to yield mean peak forces and activation lags per step cycle; the latter with respect to the first ground contact of the foot. Mean peak joint torques were approximated by multiplying mean peak forces by the corresponding moment arms as defined in Mortensen et al.’s model.

All procedures were approved by the ethics committee of the University of Magdeburg.

## Results

### Aspects of head damping control and the feedback system

During the gait cycle, the Euler analysis of the yaw and roll head movements displayed a phase shift of 180°. Using test condition 2, that shift was reduced to 146.3°; beyond that, under condition 3, that shift increased to 205°. To the best of our knowledge, this effect has not been described previously.

**Fig. 1:**
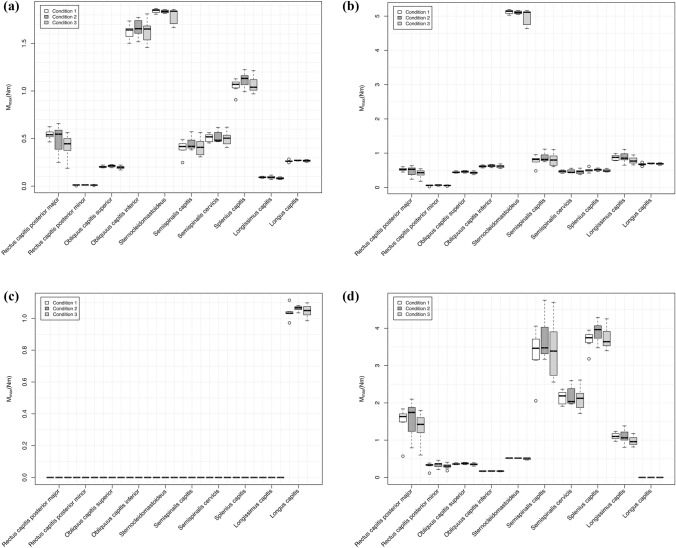
**a–d** The statistical analysis of the cervical muscle peak-torques [*M*_max_ in Newton meter (Nm)] of the volunteers while treadmill walking is demonstrated in different box plots. Statistical significances are shown in combination with the mean values (M) and standard deviation (SD) in Table 1. “Condition 1”: represents no experimental disturbance. “Condition 2”: represents a sacculus irritation. “Condition 3”: represents an electrical neck muscle irritation

The peak torque of the neck muscles might be one of the most rational parameters to demonstrate the muscular competence for head oscillation damping. Figure 1a–d represent calculated angular peak torques of the neck muscles when walking on the treadmill.

In comparison to the peak yaw torque of all other neck muscles, Fig. 1a points out that the obliquus capitis inferior muscle (≈ 1.62 Nm) and the sternocleidomastoid muscle (SCM) (≈ 1.84 Nm) were nearly the same.

Figure 1b shows that all peak roll torques of the dorsal neck muscles were below 1 Nm; only the SCM performed higher  (≈ 5.12 Nm).

Because of the complete asymmetrical pitch plane of a head–neck unit, we had to separately pitch-up and pitch-down torques. Figure 1c illustrates that only the longus capitis muscle was responsible for active pitch-down torques (≈ 1.04 Nm).

The results are different in Fig. 1d. Here, the torque of the longus capitis muscle was zero. The highest pitch-up torques were calculated in the semispinalis capitis (≈ 3.33 Nm) and splenius capitis muscles (≈ 3.68 Nm). In comparison to that, the SCM played an unexpectedly minor role in rotational pitch-up torques (≈ 0.52 Nm).

**Fig. 2:**
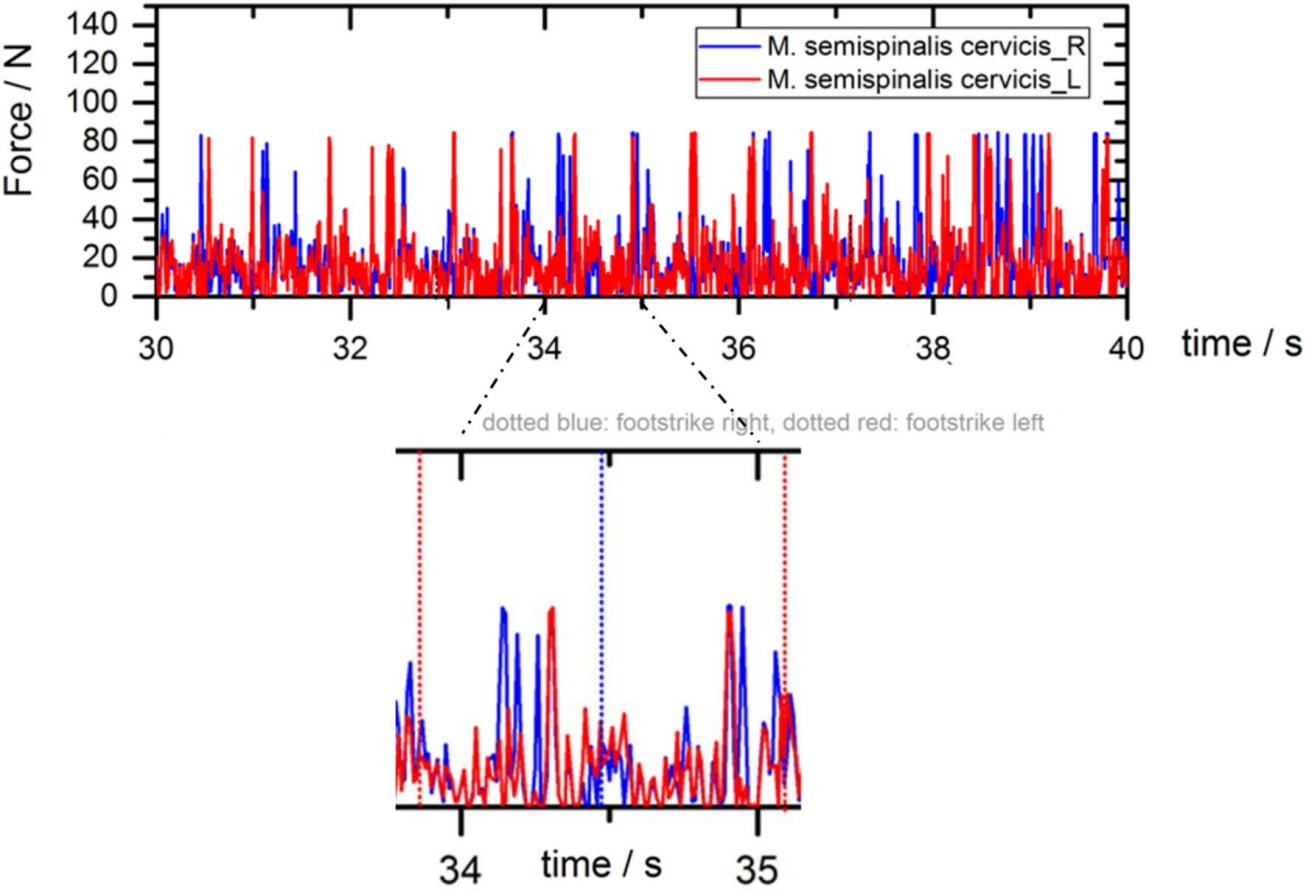
The upper figure represents the timeline of both semispinalis cervicis muscles. The lower figure is zooming in the timeline in-between the seconds of 33 up to 37. The vertical dotted line represents the foot strike. The left and right peak torques of the semispinalis cervicis muscle is shown by the red and blue lines

In relation to each foot strike, the relevant timeline of the peak torques is shown in Fig. 2 as an example of the semispinalis cervicis muscle.

All neck muscles considered to be relevant for head oscillation damping are listed in Table 1. Here, a three-dimensional movement range for each neck muscle is dedicated and underlines the complexity of its motor control (e.g., to control only six eye muscles seems to be much easier).

**Table 1:** Three-dimensional peak torques of head oscillation damping

Subject	Length (cm)^#^	Weight (g)^#^	Spindle (rel.)^#^	Peak yaw-torques			Peak roll-torques			Peak pitch-up torques			Peak pitch-down torques		
				Condition *M* (SD)			Condition *M* (SD)			Condition *M* (SD)			Condition *M* (SD)		
				1	2	3	1	2	3	1	2	3	1	2	3
Rectus capitis posterior major	5.0	4.0	2.5	0.54 (0.05)	**0**.49*** (0.**16)**	**0**.42** (0.**12)**	0.52 (0.05)	**0**.47*** (0.16)	**0**.41** (0.12)	1.74 (0.16)	**1**.55*** (0.**52)**	**1**.35** (0.**40)**	0.0 (0.0)	0.0 (0.0)	0.0 (0.0)
Rectus capitis posterior minor	3.0	1.5	1.8	0.01 (0.00)	0.01 (0.00)	0.01 (0.00)	0.06 (0.02)	0.06 (0.02)	0.06 (0.01)	0.31 (0.09)	0.34 (0.08)	0.30 (0.07)	0.0 (0.0)	0.0 (0.0)	0.0 (0.0)
Obliquus capitis superior	6.0	3.3	3.0	0.20 (0.01)	**0**.21* (0.**01)**	0.20 (0.02)	0.44 (0.02)	**0**.46* (0.**03)**	0.43 (0.04)	0.36 (0.02)	**0**.38* (0.**02)**	0.35 (0.03)	0.0 (0.0)	0.0 (0.0)	0.0 (0.0)
Obliquuus capitis inferior	5.5	7.9	3.5	1.62 (0.08)	1.66 (0.10)	1.62 (0.12)	0.61 (0.03)	0.63 (0.04)	0.61 (0.05)	0.17 (0.01)	0.17 (0.01)	0.17 (0.01)	0.0 (0.0)	0.0 (0.0)	0.0 (0.0)
Sternocleido mastoideus	15.0	53.4	1.8	1.84 (0.02)	1.84 (0.02)	**1**.78*** (0.**08)**	5.12 (0.07)	5.11 (0.05)	**4**.95*** (0.**23)**	0.52 (0.01)	0.52 (0.00)	**0**.50*** (0.**02)**	0.0 (0.0)	0.0 (0.0)	0.0 (0.0)
Semispinalis capitis	11.0	29.1	4.9	0.40 (0.08)	0.45 (0.07)	0.41 (0.10)	0.50 (0.15)	0.51 (0.06)	0.81 (0.04)	3.33 (0.65)	3.73 (0.57)	3.42 (0.79)	0.0 (0.0)	0.0 (0.0)	0.0 (0.0)
Semispinalis cervicis	10.0	n.d	n.d	0.51 (0.04)	0.52 (0.06)	0.50 (0.07)	0.46 (0.04)	0.47 (0.06)	0.45 (0.07)	2.14 (0.25)	2.19 (0.29)	2.10 (0.31)	0.0 (0.0)	0.0 (0.0)	0.0 (0.0)
Splenius capitis	11.0	29.1	1.8	1.05 (0.07)	1.12 (0.08)	1.07 (0.09)	0.88 (0.99)	0.93 (1.11)	0.77 (0.92)	3.68 (0.25)	**3**.90* (0.**29)**	3.74 (0.31)	0.0 (0.0)	0.0 (0.0)	0.0 (0.0)
Longissimus capitis	12.0	8.0	7.5	0.09 (0.01)	0.09 (0.02)	**0**.08* (0.**01)**	0.87 (0.09)	0.88 (0.16)	0.79 (0.11)	1.11 (0.10)	1.10 (0.19)	**0**.98* **(0**.**14)**	0.0 (0.0)	0.0 (0.0)	0.0 (0.0)
Longus capitis	14.0	n.d	n.d	0.26 (0.01)	0.27** (0.00)	0.27* (0.01)	0.67 (0.04)	0.70*** (0.01)	0.69 (0.03)	0.0 (0.0)	0.0 (0.0)	0.0 (0.0)	1.04 (0.04)	1.06** (0.02)	1.05 (0.04)
Overall results				6.27 (0.38)	6.38 (0.53)	6.09 (0.63)	9.19 (1.31)	9.15 (1.55)	8.29 (1.46)	26.07 (2.94)	27.74 (3.92)	25.82 (4.18)	2.08 (0.04)	2.12 (0.02)	2.10 (0.04)

The table represents a three-dimensional aspect of the calculated peak-torques of neck muscle pairs. As in the study setup described, the results of the mean values (M) and the standard deviation (SD)  are separated in condition 1 (represents no experimental disturbance), condition 2 (represents a sacculus irritation) and condition 3 (represents an electrical neck muscle irritation). Our data’s are completed by the anatomical research of Banks [38]

*p < 0.1

**p < 0.05

***p < 0.01

For statistical analysis, we performed tests comparing the mean values (paired *t* test, *α* < 0.5) and variance (*f* test, *α* < 0.5) of all neck muscle peak torques under conditions 1 and 2 and conditions 1 and 3. When comparing the variances, significant deviations are noticeable in Table 1.

The last line of the table featured a summation of all neck muscle peak torques in one movement direction. The overall results emphasise the prominence of head pitch-down damping (overall result of peak pitch-up torques ≈ 26.07 Nm).

### Aspects of head damping biomechanics

**Fig. 3:**
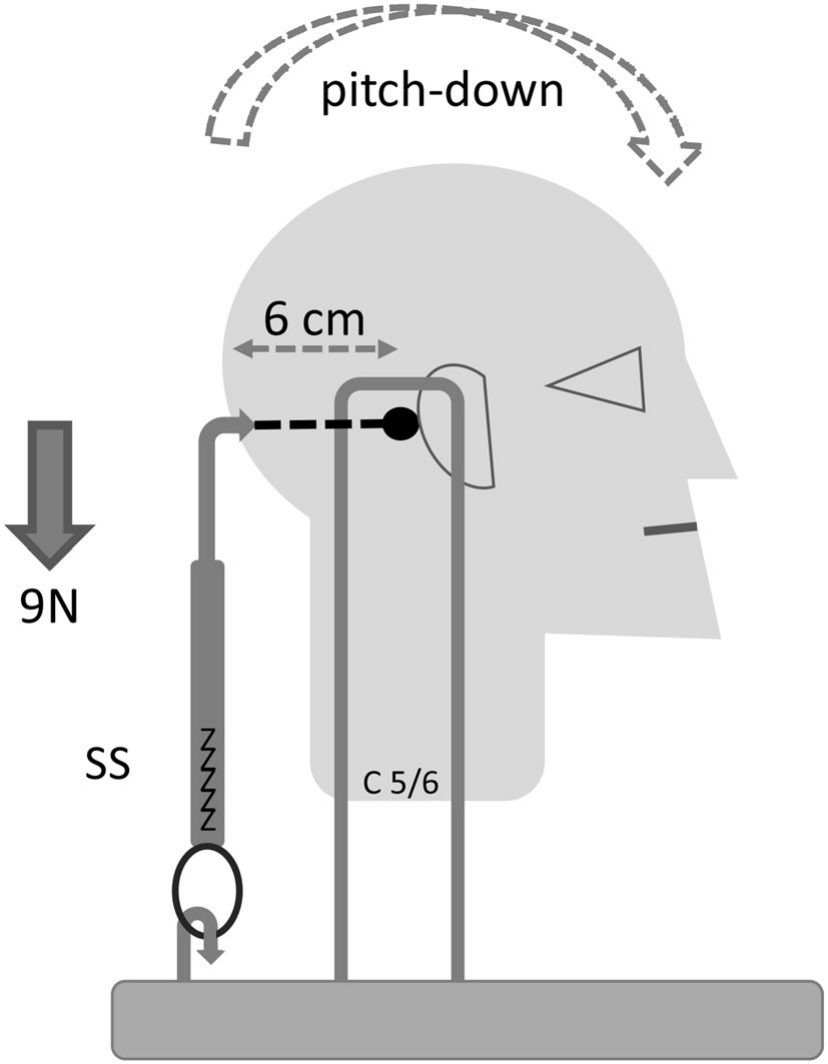
Scheme of the setup for pitch stabilizing torque measurement using an anatomical head/neck specimen. The specimen was fixed in a steel frame allowing free rotation around the pitch axis (black dot). A spring scale (ss) was fixed in the scalp of the occiput. The lever between this fixation point and the pitch axis was 6 cm. An average of 9 Newtons (N) had to be applied to prevent anterior rotation (stippled curved arrow) of the specimen. This shows that the centre of gravity of the head/neck unit in adults lies in front of the pitch axis

Finally, the neck muscular challenge in pitch damping could be demonstrated by biomechanical characteristics. If the head/neck unit of the three specimens could rotate freely around the pitch axis, the head rotation was always anterior (pitch-down). An average force of 9 N had to be applied at the occiput to keep the specimen horizontal. Calculated with a lever of 6 cm, this results in a torque of 0.54 Nm (Fig. 3). This pitch-up torque of 0.54 Nm means that the centre of gravity of the head/neck unit in adults has to be in front of the pitch axis.

## Discussion

### Vestibular control of neck muscles

During a walking cycle, a yaw–roll phase relationship of 180° means that the head oscillates continuously between a vestibular organ position of RALP (right anterior/left posterior) and LARP (left anterior/right posterior). The abbreviations RALP/LARP describe the sagittal positional alignment of the posterior semicircular canal by roll and yaw. Because of the three-dimensionally tilted position of the labyrinth within the petrosal bone, only the RALP or LARP planes provide the optimal position for the posterior semicircular canal and the sacculus to detect and control the continuous pitch movements [4].

**Fig. 4:**
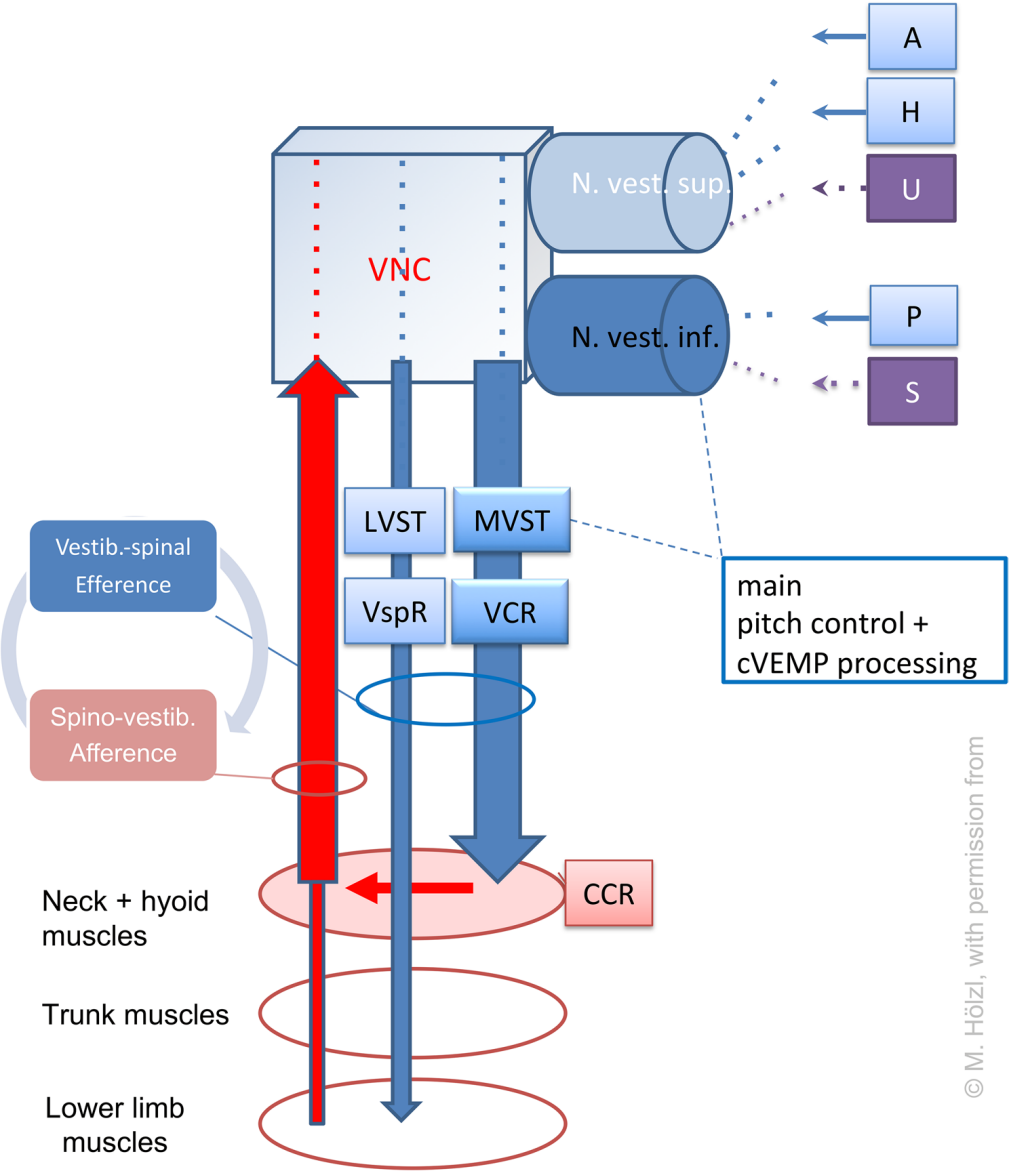
Simplified diagram of multimodal control of neck muscles for head oscillation damping during walking. Pitch acceleration is detected mainly by the sacculus (S) and posterior semicircular canal (P) projecting via the inferior vestibular nerve (yellow) to vestibular nuclei complex (VNC). Vestibular control of neck muscles is mediated mainly through the medial vestibulospinal tract (MVST) and the vestibulo-collic reflex (VCR), whereas the lateral vestibulospinal tract (LVST) and the vestibulo-spinal reaction (VspR) may play a minor role. Proprioceptive feedback from neck muscle spindles (red) feeds into the VNC and in the cervico-collic reflex (CCR). *A, H, U* anterior and horizontal semicircular canals and utriculus, respectively; *C, T, L, Sc* cervical, thoracic, lumbar and sacral spinal cord segments, resp.; *N. vest. sup., inf.* superior and inferior vestibular nerves, resp.; *cVEMP* cervical vestibular-evoked myogenic potential

The results suggest that alternating RALP and LARP head movements are precisely controlled by complex neck muscle patterns (Fig. 4).

### Separate vestibular perturbation of neck muscle function

We attribute the yaw–roll phase shift from 180° to 146.3° to artificial sacculus irritation (condition 2) during walking. This suggests that cervical vestibular evoked myogenic potential (cVEMP) may be involved in dynamic head coordination during walking. The cVEMP describes the vestibular influence on motor control of the neck muscles. Our assumption is strongly influenced by the results of Forbes et al.’s study. They systematically documented cVEMP bilaterally in the sternocleidomastoid, splenius capitis, sternohyoideus, semispinalis capitis, multifidus, rectus capitis posterior and obliquus capitis inferior [5]. The cVEMPs are also described by Goldberg and Cullen as linear vestibulocollic reflex [6]. The neuronal connections of all vestibular receptors to the cervical spinal segments were reported by Uchino et al. [7]. Their neurophysiological study results on decerebrated cats highlighted the convergence of the posterior semicircular canal with the otolithic organs’ neurons at more than 30%. This may be relevant because, especially in humans, the inferior vestibular nerve includes the neurons of only the saccular nerve and the posterior ampullary nerve [8].

Animal experiments demonstrated consistently that all five vestibular receptors on each side have specific neuronal connections to the cervical spinal motor neurons via the medial vestibulospinale tractus (MVST) [9, 10].

In humans, the MVST was first demonstrated by MRI in 2018 [11]. The MVST projects mainly to motor neurones and interneurones in the upper half of the cervical spinal cord and tapers more caudally. Interactions of vestibular influences with cortical (via the pyramidal tract) and extrapyramidal pathways may take place at cervical segments. Neurophysiological studies on decerebrated cats described the connection of MVST to the spinal motor neurons of the obliquus capitis (inferior and superior), rectus capitis posterior, splenius capitis and semispinalis capitis muscles [12, 13]. Sugiuchi et al. concluded that these muscles assist the stabilisation of the head and that their inhibitory neurons are located in the cervical spinal cord [14].

Conclusions from such experimental animal findings can be applied only with some caution to active vestibular everyday tasks (e.g., walking on a treadmill). In 2008, Xiang et al. studied head stabilisation in monkeys on the treadmill [15]. Their study results on non-human primates were consistent with the results on decerebrated cats. Sadeghi et al. concluded in humans with semicircular canal occlusions, that a direct sensorimotor transmission into the upper cervical medulla must occur [16].

Peng et al. supposed that this kind of transmission is used for head oscillation damping during gait and may be interpreted as a main undertaking of the vestibulocollic reflex (VCR) [17]. Current reviews have underlined the importance of the VCR [5, 6, 18]. Moreover, some authors have assigned great of importance to the vestibulocollic reflex, especially regarding the pitch-VCR [18, 19].

To characterise the VCR, experiments were performed with volunteers [20–22]. Results showed that gait speed and age can influence head stabilisation [23–25]. Significant loss in head stabilisation was demonstrated in patients with unilateral and bilateral vestibular dysfunction [26–28].

The control of the neck muscles is not based solely on vestibular signals. The frequently used term “sensorimotor control” is mainly due to the mutual spinal influences of visual and vestibular pathways. Further influences occur via reticulospinal pathways that can also be controlled via the interstitial nucleus of Cajal. The postural system can realise central efferency copies and autonomous spinal feed-forward motion patterns (reviewed [6]).

### Separate perturbation of neck muscle function

TENS is a widely established electrical device to alter the excitability of peripheral nociceptors to reduce afferent input to the central nervous system. With low frequency stimulation (30 Hz), no motor impairments of muscle functions have yet been reported [29]. Corresponding to the well-known discriminating impairment of only nociceptors of TENS, we interpreted that the yaw–roll phase shift from 180° to 205° might be a result of disturbed proprioceptors (condition 3). The integration of neck muscle proprioceptive and vestibular signals converging in vestibular nuclei has been frequently studied. There is good evidence that neck muscle proprioception forms the afferent limb of the cervicocollic reflex (CCR; review [18]). The CCR is understood as a stretching reflex that recruits other groups of neck muscles during cervical stretching (co-contraction) [17]. The CCR is in close coordination with the VCR (reviewed [30]). Forbes et al. found that the two neck reflexes are responsible for head stabilisation [31]. In 2017, this group described relevant reflex control strategies for VCR and CCR using a multi-segment cervical spine model [32].

Neck muscles are known for their richness of muscle spindles [33]. Spindle afferents project not only to the motor neuronal and interneuronal pools in the ventral horn of the cervical spinal cord, but also both directly and indirectly via the central cervical nucleus (CCN) to vestibular nuclei [35]. The integration of labyrinthine and neck muscle afferents in vestibulospinal neurons has been extensively documented [34]. On the other hand, the CCN receives labyrinthine input via vestibular nuclei [35]. Thus, there are multiple levels of integration of labyrinthine and neck proprioceptive afferents. These connections support the assumption that the proprioceptive feedback loop of the VCR can adaptively modify head coordination.

Mulavara et al. emphasised the importance of the somatosensory system for head stabilisation by investigating cosmonauts in environmental microgravity [36]. Our results are in accordance with their conclusion that somatosensory influences adaptively modify head movement control during locomotion.

Muscle vibration is recognised as an artificial proprioceptive disorder (reviewed [37]). Bove et al. reported that experimental vibrations of the sternocleidomastoid muscle (SCM) are associated with a gait deviation to the opposite side [38, 39]. Our results of the SCM peak yaw torques may rationalise these findings once more (Fig. 1). In continuous neck vibrations on the treadmill, Ivanenko showed significant changes in postural reference during quiet standing and in walking speed during locomotion [40].

Vuillerme et al. concluded that the vestibular system receives disrupted proprioceptive information under muscle vibration [41].

A pathophysiological disorder of proprioception includes the concept of muscle fatigue. Numerous studies have shown that once muscle fatigue sets in, the muscle is no longer able to adequately transmit somatosensory information [42, 43].

However, the authors argue that it might be possible that the artificial proprioceptive perturbation performed in our condition 3 disturbed the feedback loop of the VCR. Thus, one effect of the yaw–roll phase shift might be interpreted as a result of disturbed neck muscle proprioception.

### Biomechanics

Muscular head oscillation damping mechanisms required for the position of the CG of the head–neck unit. Of the three planes, only the pitch plane has a lack of symmetry between the two sagittal halves. It is not clear whether the ventral soft tissue mass, which is attached to the viscerocranium, is comparable to the dorsal soft tissue mass. Our anatomical results argue for a ventral centre of the gravity position of the head–neck unit. A torque of 0.54 Nm is required at rest to hold the horizontal position of our specimen. Without constant activity of the neck musculature, the head would fall on the chest during rest (e.g., a seminar participant falling asleep).

For the model of the human gait, this means that across all movement dimensions to be stabilised, the pitch-down head movement is the most unstable and critical momentum. When walking, the swinging leg constantly causes positive and negative acceleration forces on the head. To dampen the pitch-down head movement, the dorsal neck muscles must be eccentrically active. The calculations of our OpenSim software show that under our setup conditions, a total torque of 26.07 Nm is available to dampen the pitch-down oscillation (Table 1). The comparatively lower torques for roll and yaw oscillation accentuate the challenge of pitch-down oscillation damping. Moreover, a static horizontal head position is alone a function of the muscular head control system, which helps to support optimal functioning of all our sensory organs.

The competence of the individual neck muscles for head oscillation damping can by no means be derived intuitively. As an example, the sternocleidomastoid muscle (SCM) could be singled out. The SCM is generally known as the large neck rotation muscle (yaw axis). However, under the study conditions, the maximum torque of the obliquus capitis inferior muscle is almost identical to that of the SCM (Fig. 1), irrespective of the anatomical difference between the two muscles.

It appears that the pitch-up oscillation may have a cascade damping mechanism. Up to a pitch torque of about 0.54 Nm, the head oscillation damping requires no cervical muscles because, first, the negative ventral torque supports the oscillation damping in the pitch-up direction. Second, the torque of the ventral inner neck muscles is remarkably low (≈ 2.08 Nm; Table 1). It is interesting to note that Forbes et al. (in the sternohyoid muscle [5]) and De Natale et al. (in the masseter muscle [44]) provided normative data of VEMPs in the hyoid muscle group. Mortensen et al. pointed to the importance in the context of head stabilisation [45]. We do interpret this as a further indication that the vestibular system could also antagonistically recruit the hyoid muscle groups, resulting in stronger pitch-up accelerations (e.g., header in soccer).

The head oscillation damping is not based solely on the neck muscles. There is evidence that during walking, the regulation of thoracic spine movement may help stabilise the head’s CG [20, 23, 46]. Lee et al. reported similar results for the cervical spine [47].

### Limitations

There are some limitations in the present study. As our study model is based on the published head–neck muscle work of Mortensen et al. [4], our model is subject to the same limitations. Furthermore, human head–neck muscles show high interpersonal and intrapersonal variations, so every numeric model can be only a simplified model. The adaptation of muscle lengths to the individual stature is necessary and an improvement to earlier models, but a solely linear adaptation cannot represent physiological inner muscle conditions. The biomechanical influence of other soft tissue, such as fascia or the cutis, could not be integrated due to a lack of knowledge of their exact biomechanical influence on the gait and posture. Our anatomical study used a cadaver of an older human. For an exact interpretation, it would be reasonable to use a statistically relevant number of cadavers of different ages, genders and constitutions. Also, for the experimental study on the treadmill, further research with a high number of healthy volunteers is necessary.

## Conclusion

Regarding the actual literature and our study results we hypothesize a multimodal control of neck muscles for head oscillation damping (Fig. 4). Under study conditions, we observed multimodal neck muscle functions in head damping:

1. Vestibular associated control system
  - oscillation damping of the head
  - coordination of the RALP/LARP positions
2. Proprioceptive associated feedback system
  - for proprioceptive feedback of the vestibulocollic reflex (VCR)
  - triggering the cervicocollic reflex (CCR).

From a clinical point of view, this pilot study reveals perspectives suggesting that head damping’s feedback system could be of clinical relevance in, for example, whiplash-associated symptoms or in all aspects of so called “cervical vertigo” [48, 49] or “postural imbalance” [50].

Further research is needed.

## Abbreviations

Cranial pitch angle: Head flexion/extension
Cranial roll angle: Head lateral flexion/extension
Cranial yaw angle: Head transversal rotation
VEMP: Vestibular elicited myogenic potential
vHIT: Video head impulse test
TENS: Transcutaneous electric nerve stimulation
RALP: Vestibular organ position of right anterior/left posterior
LARP: Vestibular organ position of left anterior/right posterior.
MVST: Medial vestibulospinale tractus
LVST: Lateral vestibulospinale tractus
VCR: Vestibulocollic reflex
CCR: Cervicocollic reflex
CCN: Central cervical nucleus
SCM: Sternocleidomastoid muscle
